# Adaptive magnetic resonance-guided neurovascular-sparing radiotherapy for preservation of erectile function in prostate cancer patients

**DOI:** 10.1016/j.phro.2021.09.002

**Published:** 2021-09-21

**Authors:** Frederik R. Teunissen, Ruud C. Wortel, Jochem Hes, Thomas Willigenburg, Eline N. de Groot-van Breugel, Johannes C.J. de Boer, Harm H.E. van Melick, Helena M. Verkooijen, Jochem R.N. van der Voort van Zyp

**Affiliations:** a Department of Radiation Oncology, University Medical Center Utrecht, Utrecht, The Netherlands; b Department of Urology, University Medical Center Utrecht, Utrecht, The Netherlands; c Department of Urology, St. Antonius Hospital, Nieuwegein, Utrecht, The Netherlands; d Imaging and Oncology Division, University Medical Center Utrecht, Utrecht, The Netherlands; e Utrecht University, Utrecht, The Netherlands

**Keywords:** MRgRT, Localized prostate cancer, MR-Linac, Neurovascular-sparing, Erectile function sparing, Erectile dysfunction

## Abstract

**Background and purpose:**

Erectile dysfunction is a common adverse effect of external beam radiation therapy for localized prostate cancer (PCa), likely as a result of damage to neural and vascular tissue. Magnetic resonance-guided online adaptive radiotherapy (MRgRT) enables high-resolution MR imaging and paves the way for neurovascular-sparing approaches, potentially lowering erectile dysfunction after radiotherapy for PCa. The aim of this study was to assess the planning feasibility of neurovascular-sparing MRgRT for localized PCa.

**Materials and methods:**

Twenty consecutive localized PCa patients, treated with standard 5×7.25 Gy MRgRT, were included. For these patients, neurovascular-sparing 5×7.25 Gy MRgRT plans were generated**.** Dose constraints for the neurovascular bundle (NVB), the internal pudendal artery (IPA), the corpus cavernosum (CC), and the penile bulb (PB) were established. Doses to regions of interest were compared between the neurovascular-sparing plans and the standard clinical pre-treatment plans.

**Results:**

Neurovascular-sparing constraints for the CC, and PB were met in all 20 patients. For the IPA, constraints were met in 19 (95%) patients bilaterally and 1 (5%) patient unilaterally. Constraints for the NVB were met in 8 (40%) patients bilaterally, in 8 (40%) patients unilaterally, and were not met in 4 (20%) patients. NVB constraints were not met when gross tumor volume (GTV) was located dorsolaterally in the prostate. Dose to the NVB, IPA, and CC was significantly lower in the neurovascular-sparing plans.

**Conclusions:**

Neurovascular-sparing MRgRT for localized PCa is feasible in the planning setting. The extent of NVB sparing largely depends on the patient’s GTV location in relation to the NVB.

## Highlights

• Magnetic resonance-guided radiotherapy enables visualization and dose adaptation of neurovascular structures.
• Neurovascular-sparing magnetic resonance-guided radiotherapy hypothetically preserves erectile function.
• Neurovascular-sparing 5×7.25 Gy magnetic resonance-guided radiotherapy for localized prostate cancer is feasible.
• Extent of neurovascular bundle sparing largely depends on tumor location.

## 1 Introduction

Erectile dysfunction (ED) is a common adverse effect of external beam radiation therapy (EBRT) for localized prostate cancer (PCa). In patients treated with stereotactic body radiation therapy (SBRT), ED rates range from 26% to 55% at 60 months in previously sexually functioning patients [1]. The prostate is surrounded by structures responsible for the erectile function such as the neurovascular bundles (NVBs), the internal pudendal arteries (IPAs), the corpora cavernosa (CCs), and the penile bulb (PB). Radiation damage to these structures potentially leads to a decline of erectile function after treatment [2].

Neurovascular-sparing radiotherapy for erectile function preservation has been proposed before [2], [3], [4]. Spratt et al. reported the first vessel-sparing treatment trial, delivering EBRT to the prostate while sparing the IPAs and CCs [5]. Their results were promising with a reported erectile function preservation rate of 67% (i.e. International Index of Erectile Function (IIEF)-5 ≥ 16) at 5 years after treatment [6]. Currently, the POTEN-C trial is ongoing, aiming to preserve erectile function in patients with localized PCa by sparing the NVBs, IPAs, and PB using a conventional linac system to deliver SBRT in 5 fractions of 8–9 Gy [7].

Due to the movement of the pelvic organs, daily plan optimization is desirable for neurovascular-sparing radiotherapy. However, NVBs and IPAs cannot be adequately identified on CT due to lack of contrast. MRI allows better visualization of these structures [2], [4]. Therefore, magnetic resonance-guided online adaptive radiotherapy (MRgRT) could pave the way for an optimized neurovascular-sparing approach. MR imaging prior to and during dose delivery facilitates correction for interfraction motion and tissue deformations. Within the near future fast auto contouring and planning will provide a way to deal with intrafraction motion, thus allowing for further margin reduction and reduction of dose to organs at risk (OAR) [8], [9].

To date, no study has examined the planning feasibility of neurovascular-sparing MRgRT for localized PCa. Therefore, in this study we aimed to assess the feasibility of treatment planning for neurovascular-sparing MRgRT for localized PCa and the potential dose reduction to neurovascular structures.

## 2 Materials and methods

### 2.1 Patient characteristics

For this planning study, 20 consecutive patients with localized low- to high-risk PCa (National Comprehensive Cancer Network (NCCN) risk categories) without extracapsular extent were included, to account for the variation in tumor location and anatomy of the localized PCa population. All patients were previously treated with standard 5×7.25 Gy MRgRT on a Unity MR-Linac. In preparation for treatment on the MR-Linac patients received a pre-treatment multiparametric (mp) 3T offline planning MRI (T2-weighted and diffusion weighted imaging (DWI) sequences; reconstructed resolution (mm^3^): 0.8/0.8/2.0) for optimal contouring of target volumes and OAR. Patients signed informed consent for sharing of their clinical data within the MOMENTUM study (NCT04075305), which was approved by our institutional review board [10], [11].

### 2.2 Neurovascular-sparing dose constraints and volume definitions

Dose constraints for the NVB, IPA, CC, and PB for a 5-fraction scheme were established by consensus of a board of 4 expert prostate specialized radiation oncologists (25, 15, 10, and 10 years of clinical experience, respectively) and a radiation biologist (Supplementary Material 1). For neurovascular tissue an EQD2 α/β of 2.0 Gy and for vascular tissue an α/β of 3.0 Gy was applied [12]. The constraints for the IPA and CC were based on the 5-fraction equivalent of the constraints as used in the study by Spratt et al. (IPA 100% < 36.0 Gy, CC 100% < 30.0 Gy in 42 fractions) [5]. The PB constraint was based on the PACE-trial constraint (D50% < 29.5 Gy in 5 fractions) [13], [14]. For the NVB no constraints were described in literature. Therefore, the NVB dose constraint was based on literature for neural and vascular tissue and experience with radiation toxicity for both sacral and brachial plexus and was set to D0.1 cc ≤ 32.8 Gy [5], [14], [15], [16].

The GTV + 4 mm included the GTV (mpMRI visible tumor(s)) with a 4 mm isotropic margin excluding the rectum and bladder. The CTV included the GTV + 4 mm and prostate body with the base of the seminal vesicles and the PTV included the CTV with a 5 mm isotropic margin. Dose prescriptions for the PTV were adapted to allow neurovascular-sparing MRgRT. The GTV + 4 mm should receive 34.4 Gy in ≥ 99% and the PTV 30.0 Gy in ≥ 99%; 32.6 Gy in ≥ 90% and 34.4 Gy in ≥ 80%. Because of the proximity of the NVB to the prostate and the priority of dose coverage of the GTV + 4 mm and PTV, we set the NVB dose constraint as “soft” constraint (i.e. not mandatory). The applied dose constraints for neurovascular-sparing 5×7.25 Gy MRgRT are displayed in Table 1.

**Table 1:** Target volume dose prescription and dose constraints for neurovascular-sparing 5×7.25 Gy MRgRT.

Structure	Parameter	Dose constraint	
		Soft	Hard
PTV	V34.4 Gy (V95%)		≥ 80.0%
	V32.6 Gy (V90%)		≥ 90.0%
	V30.0 Gy (V83%)		≥ 99.0%
GTV + 4 mm	V34.4 Gy (V95%)		≥ 99.0%
Bladder	D0.5 cc		< 42.0 Gy
	D5 cc		< 37.0 Gy
	V32.0 Gy		< 15.0%
	V28.0 Gy		< 20.0%
Femur	D10 cc	< 30.0 Gy	
Rectum	D0.5 cc		≤ 40.0 Gy
	D1 cc	≤ 35.0 Gy	≤ 38.0 Gy
	V32.0 Gy		≤ 15.0%
	V28.0 Gy		≤ 20.0%
Sphincter (distal 3 cm of rectum)	D0.5 cc		≤ 40.0 Gy
	D1 cc	≤ 35.0 Gy	≤ 38.0 Gy
	Dmean		< 20.0 Gy
NVB	D0.1 cc	≤ 32.8 Gy	
IPA	D0.1 cc		≤ 20.0 Gy
CC	D0.01 cc		≤ 17.3 Gy
PB	D50%		< 29.5 Gy

Abbreviations: GTV = gross tumor volume; PTV = planning target volume; NVB = neurovascular bundle; IPA = internal pudendal artery; CC = corpus cavernosum; PB = penile bulb.

### 2.3 Neurovascular-sparing treatment planning

**Fig. 1:**
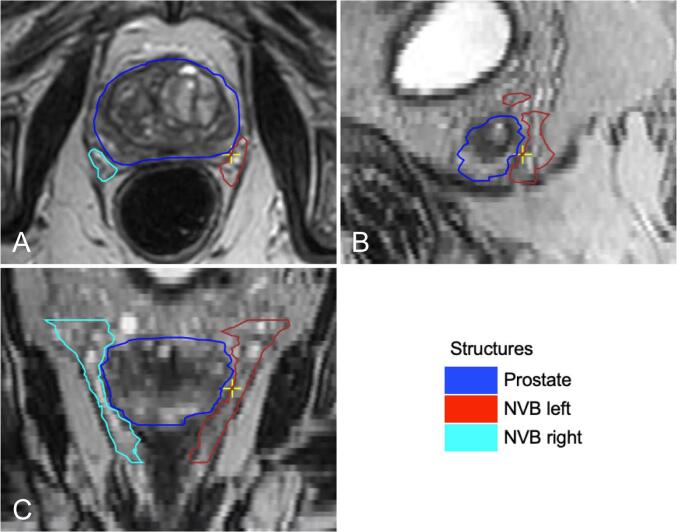
Example of the contours of the neurovascular bundle of a single study patient. Abbreviations: NVB = neurovascular bundle. A: transverse plane; B: sagittal plane; C: coronal plane.

For each patient the left and right NVB, IPA, CC, and the PB were contoured on the pre-treatment offline 3T T2-weighted planning MRI. Contouring was done by a single prostate specialized radiation oncologist (JVZ) with 10 years of clinical experience, using the in-house developed contouring software package Volumetool and contours were added to the standard planning contour set that was previously contoured by the treating radiation oncologist. The NVB was contoured from at least the base of the seminal vesicles until the level of the urogenital diaphragm (Fig. 1). On T2-weighted MRI the NVB is generally well identifiable at the level of the apex where it is delimited by the dorsolateral part of the prostate and the ventrolateral part of the rectum and can be followed towards the level of the seminal vesicles [2], [17]. The IPA was contoured from at least the level of the sacroiliac ligament until the crus where it terminates into the common penile artery and the scrotal artery.

The planning MRI including contour set was imported into the treatment planning software Monaco 5.40.01 (Elekta AB, Stockholm, Sweden), to generate intensity modulated radiation therapy (IMRT) offline treatment plans for the Unity MR-Linac. Bulk relative electron density value of 1 was assigned to the body and values for the femoral heads and other bony structures were calculated using the average Hounsfield units of a matched CT scan. Seven-field IMRT technique was used (gantry angles: 0°, 50°, 100°, 155°, 205°, 260°, and 310°). The calculation grid spacing was 3 mm with a statistical uncertainty of 3% per control point and < 1% per voxel. The minimum segment width was 0.5 cm and area 1.5 cm^2^ and the minimum number of motor units was 5 with a maximum of 60 segments. No plan renormalization was used. During treatment the patient is supported by a soft pillow under the head and knee supporters under the feet. All settings were identical to the standard 5×7.25 Gy MRgRT at our institution.

For neurovascular-sparing treatment planning, GTV + 4 mm and PTV coverage was the primary goal, secondary, meeting the conventional OAR (bladder, rectum, sphincter, and femurs) constraints, and tertiary, meeting the neurovascular structures constraints. In case the neurovascular-sparing constraints could not be met, a dose as low as reasonably achievable (ALARA) was pursued. The planning was done under supervision of a radiation therapist specialized in treatment planning (JH) with 10 years of clinical experience and all plans were evaluated by a prostate specialized radiation oncologists (JVZ).

### 2.4 Plan comparison

In a next step, we compared the neurovascular-sparing plans with the standard (i.e. non-neurovascular-sparing) pre-treatment plans. For all 20 patients, the matched neurovascular-sparing contour set including the NVBs, IPAs, CCs, and PB was registered to the actual clinical pre-treatment plan that was generated before to the MR-Linac treatment, using Monaco 5.40.01. Planned dose to the target volumes, conventional OAR, and neurovascular structures as would have been received in the standard planning setting were calculated in Monaco.

### 2.5 Statistical analysis

R version 4.0.5 was used for the statistical analysis. Pairwise Wilcoxon signed rank tests with Bonferroni correction for multiple testing were performed to compare the neurovascular-sparing planned dose with the standard planned dose. Furthermore, the NVBs were stratified between those that did and did not meet the dose constraint in the neurovascular-sparing plans. Population-median dose volume histogram (DVH) curves were generated using the R package “dvhmetrics”. Non-normally distributed data were presented as median with range and p-value of < 0.05 was considered statistically significant.

## 3 Results

**Fig. 2:**
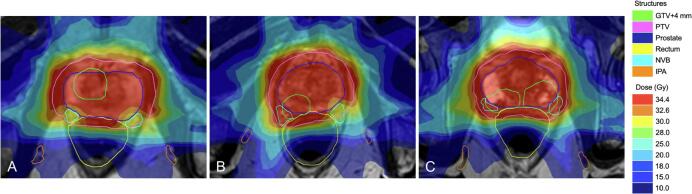
Example of neurovascular-sparing 5×7.25 Gy MRgRT plan dose distribution in 3 patients representing the 3 neurovascular bundle-sparing scenarios. Abbreviations: GTV = gross target volume; PTV = planning target volume; NVB = neurovascular bundle; IPA = internal pudendal artery. A: NVB constraint met bilaterally (40% of patients); B: NVB constraint met unilaterally (40% of patients); C: NVB constraint not met (20% of patients).

All 20 patients’ treatment plans were considered clinically acceptable. Prescribed dose coverage of the GTV + 4 mm and PTV was achieved for neurovascular-sparing plans and the neurovascular-sparing dose constraints for the CC and PB were met in all patients (Table 2). The dose constraints for the IPA were met in 19 (95%) patients bilaterally and in 1 (5%) patients unilaterally. Constraints for the NVB were met in 8 (40%) patients bilaterally, in 8 (40%) patients unilaterally, and were not met in 4 (20%) patients (Fig. 2). In all cases where the GTV was located in the dorsolateral position, the NVB constraint could not be met.

**Table 2:** Median prescribed dose for the neurovascular-sparing 5×7.25 Gy MRgRT plans compared to the standard 5×7.25 Gy MRgRT pre-treatment plans.

Structure	Volume (cc)		Planned dose					
			Parameter	Neurovascular-sparing plans		Standard plans*		p
	median	range		median	range	median	range	
PTV (n = 20)	105.8	68.3–185.2	V34.4 Gy (%)	88.3	81.1–97.4	98.9	97.0–99.9	< 0.01
			V32.6 Gy (%)	95.8	90.8–99.9	99.9	98.6–100	< 0.01
			V30.0 Gy (%)	99.9	99.5–100	100	99.2–100	0.03
GTV + 4 mm (n = 20)	14.1	1.6–57.9	V34.4 Gy (%)	100	99.8–100	100	100–100	< 0.01
Bladder (n = 20)	183.9	71.1–408.3	D0.5 cc (Gy)	37.2	36.5–37.9	37.1	35.1–38.3	0.03
			D5 cc (Gy)	35.7	34.3–36.8	35.7	33.1–36.8	0.71
			V32.0 Gy (%)	9.8	3.7–13.9	8.9	2.6–18.5	0.99
			V28.0 Gy (%)	15.3	5.9–20.0	13.8	4.4–27.5	0.99
Femur (n = 40)	217.5	146.7–290.6	D10 cc (Gy)	17.9	12.0–20.9	16.9	13.3–20.5	0.13
Rectum (n = 20)	79.9	50.6–190.8	D0.5 cc (Gy)	36.3	35.3–37.2	37.2	36.5–37.9	< 0.01
			D1 cc (Gy)	35.8	34.8–36.7	36.8	36.0–37.6	< 0.01
			V32.0 Gy (%)	6.1	3.3–9.2	7.7	4.9–13.1	< 0.01
			V28.0 Gy (%)	9.7	5.8–14.3	11.1	7.2–19.0	< 0.01
Sphincter (n = 20)	12.9	6.2–17.8	D0.5 cc (Gy)	24.0	2.9–34.9	24.5	4.7–36.1	0.27
			D1 cc (Gy)	18.8	2.6–33.2	19.6	3.9–34.9	0.46
			Dmean (Gy)	8.5	1.7–18.4	9.0	2.3–16.0	0.99
NVB (n = 40)	5.2	2.7–8.0	Dmean (Gy)	28.5	21.6–32.8	33.3	27.1–35.5	< 0.01
			D0.1 cc (Gy)	32.6	32.3–37.3	37.5	36.9–38.3	< 0.01
NVB constraint met in NS plan (n = 24)	4.9	2.7–8.0	Dmean (Gy)	27.6	21.6–30.2	33.2	27.1–35.5	< 0.01
			D0.1 cc (Gy)	32.6	32.3–32.7	37.3	36.9–38.2	< 0.01
NVB constraint not met in NS plan (n = 16)	5.7	3.7–7.3	Dmean (Gy)	30.0	25.0–32.8	33.7	29.2–35.3	< 0.01
			D0.1 cc (Gy)	36.0	34.8–37.3	37.6	36.9–38.3	< 0.01
IPA (n = 40)	2.1	1.2–4.0	Dmean (Gy)	11.9	7.1–15.4	19.0	9.1–28.3	< 0.01
			D0.1 cc (Gy)	19.4	10.2–22.9	29.5	11.6–35.2	< 0.01
CC (n = 40)	4.6	2.4–11.0	Dmean (Gy)	4.7	1.7–9.6	5.7	1.9–13.7	< 0.01
			D0.01 cc (Gy)	13.6	3.4–17.1	19.6	4.1–32.7	< 0.01
PB (n = 20)	6.2	3.8–11.8	Dmean (Gy)	7.9	2.2–17.4	8.4	2.1–27.0	0.08
			D50% (Gy)	4.6	2.1–17.0	3.8	1.5–28.3	0.74

Abbreviations: PTV = planning target volume; GTV = gross tumor volume; NVB = neurovascular bundle; NS = neurovascular-sparing; IPA = internal pudendal artery; CC = corpus cavernosum; PB = penile bulb.

For the femur, NVB, IPA, and CC: left and right side are combined.

*Standard 5×7.25 Gy MRgRT dose prescription to PTV was 34.4 Gy in ≥ 99.0% (no separate clinical target volume or GTV + 4 mm prescription). Dose constraints for bladder, rectum, sphincter, and femur were identical for the neurovascular-sparing and standard plans.

**Fig. 3:**
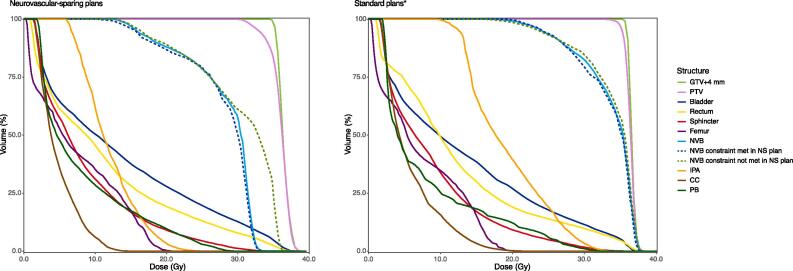
Population-median DVH curves for the neurovascular-sparing 5×7.25 Gy MRgRT plans (n = 20) and the standard 5×7.25 Gy MRgRT plans (n = 20). Abbreviations: PTV = planning target volume; GTV = gross tumor volume; NVB = neurovascular bundle; NS = neurovascular-sparing; IPA = internal pudendal artery; CC = corpus cavernosum; PB = penile bulb. Femur, NVB, IPA, and CC: n = 40 (left and right side are combined); NVB constraint met in NS plan: n = 24; NVB constraint not met in NS plan: n = 16. *Standard 5×7.25 Gy MRgRT dose prescription to PTV was 34.4 Gy in ≥ 99.0% (no separate clinical target volume or GTV + 4 mm prescription). Dose constraints for bladder, rectum, sphincter, and femur were identical for the neurovascular-sparing and standard plans. Population-median DVH curves with 95% confidence intervals are displayed in Supplementary Material 2.

The comparison of the neurovascular-sparing plans with the standard plans is presented in Table 2, Fig. 3, and Supplementary Material 2. The median planned dose to the NVB, IPA, and CC was significantly lower in the neurovascular-sparing plans compared with the standard plans (NVB D0.1 cc: 32.6 Gy vs. 37.5 Gy, p < 0.01; IPA D0.1 cc 19.4 Gy vs. 29.5 Gy, p < 0.01; CC D0.01 cc: 13.6 Gy vs. 19.6 Gy, p < 0.01), also for the cases in which the NVB constraint was not met in the neurovascular-sparing plan (D0.1 cc 36.0 Gy vs. 37.6 Gy, p < 0.01). The median planned dose to the PB was not significantly different between the neurovascular-sparing plans and the standard plans. Median dose coverage of the PTV for the V34.4 Gy parameter was significantly higher in the standard plans compared with the neurovascular-sparing plans (98.9% vs. 88.3%, p < 0.01). The median planned dose to the bladder and sphincter was not significantly different between the two planning strategies except for the bladder D0.5 cc parameter, which was significantly lower in the standard plans compared with the neurovascular-sparing plans (37.1 Gy vs. 37.2 Gy, p = 0.03). The median planned dose to the rectum was significantly lower in the neurovascular-sparing plans compared with the standard plans for all parameters (all p < 0.01).

## 4 Discussion

This study is the first to demonstrate that neurovascular-sparing MRgRT for localized PCa is feasible in the planning setting. Predefined constraints for the CC and PB were met in all 20 patients, for the IPA in 19 (95%) patients bilaterally and 1 (5%) unilaterally, and for the NVB in 8 (40%) patients bilaterally and in 8 (40%) patients unilaterally. Dose to the NVB, IPA, and CC was reduced significantly, without substantially increasing dose to the bladder, rectum, and sphincter.

In all cases where the GTV was located in the dorsolateral position of the prostate and therefore the GTV + 4 mm directly bordering or partially overlapping the NVB, the NVB constraint could not be met (Fig. 2). Nevertheless, the median planned dose to the NVBs that did not meet the dose constraint in the neurovascular-sparing plans was still lower in the neurovascular-sparing plans compared to the standard plans. In the single case where the IPA dose constraint could only be met unilaterally, the IPA had an unfavorable anatomical location close to the prostate and was therefore partly located within the PTV. It should be noted that we used an isotropic PTV margin of 5 mm for this planning study, which generally includes part of the NVB and in some cases part of the IPA. In the near future fast-adaptive auto-contouring and online re-planning will enable further margin reduction, which should improve neurovascular-sparing capabilities of MR-Linac treatment, especially the sparing of the NVB [22], [23], [24].

Although it is hypothesized that MRgRT offers major advantages in terms of erectile function sparing treatment because of the ability to adequately visualize the neurovascular structures and correct for interfraction and intrafraction motion and deformation, others have initiated studies on erectile function sparing radiotherapy on conventional linacs. Spratt et al. conducted a single arm study in which 135 men with an IIEF-5 score of ≥ 16 at baseline underwent IPA and CC sparing radiotherapy and reported an erectile function preservation rate of 67% (i.e. IIEF-5 ≥ 16) at 5 years after treatment [5]. Their study population consisted of low-, intermediate-, and high-risk PCa patients and treatment consisted of IMRT of 75.6 Gy in 1.8-Gy daily fractions or low-dose rate (LDR) brachytherapy to a prescription dose of 110 Gy, followed by IMRT of 45 Gy in 1.5-Gy fractions. For all high-risk patients, pelvic lymph nodes were treated to 45 Gy. Additionally, androgen deprivation therapy was prescribed for a duration of 6 months at the discretion of the treating physician. Because of the heterogeneity of the study population and treatment strategies, the independent effect of the different study parameters on preservation of erectile function are difficult to deduct from this study.

The currently ongoing POTEN-C trial takes erectile function sparing EBRT a step further by conducting a randomized controlled trial randomizing 120 low- to intermediate-risk patients between 5 fraction SBRT with or without sparing of the NVBs, IPAs and PB on a conventional linac [7]. The study is expected to complete in 2024. Still, to date the question remains to what extent the neurovascular structures, especially the NVB can be sufficiently and safely spared without adaptive MR-guidance.

There are some considerations for our study. First, it is unknown to what extent radiation damage to each individual neural or vascular structure contributes to ED after radiotherapy. In literature the NVBs, IPAs, CCs, and PB are generally described as the most important structures contributing to radical PCa treatment-induced ED [2], [18]. It is hypothesized that ED after radiotherapy is predominantly a consequence of vascular damage to the IPAs, CCs, PB, and vascular part of the NVBs [19]. On the other hand, ED after prostatectomy is considered primarily a consequence of nerve damage and erectile function sparing radical prostatectomy is focused on the sparing of the NVBs [20]. In literature it is widely reported that even unilateral sacrifice of the NVB will substantially increase the chance of developing ED after surgery [21]. For brachytherapy a high rate of ED after treatment is reported as well [21]. With brachytherapy, the NVBs will receive a higher radiation dose compared to the IPAs, CCs, and PB as these structures are better spared due to the typical steep dose gradient, suggesting that dose to NVBs plays an instrumental role in development of ED after brachytherapy [22]. Prospective dose-toxicity relationship studies need to be performed to adequately assess to what extent radiation damage to each individual neural or vascular structure contributes to ED after radiotherapy. Second, in this study the GTV + 4 mm was set to receive 34.4 Gy in ≥ 99.0% (EQD2 α/β = 1.5 Gy: 82.5 Gy) and the PTV 30.0 Gy in ≥ 99.0% (EQD2 α/β = 1.5 Gy: 64.3 Gy), 32.6 Gy in ≥ 90.0% (EQD2 α/β = 1.5 Gy: 74.8 Gy), and 34.4 Gy in ≥ 80.0%. These PTV constraints were lower compared to the standard 5×7.25 Gy MRgRT PTV constraint (34.4 Gy in ≥ 99.0%) used in our institution, which resulted in a significantly lower PTV dose coverage compared to the standard plans. This dose reduction might influence biochemical control, but an increase of clinical failure and decrease of overall survival is not expected. A dose escalation study from 68 Gy to 78 Gy in mainly high-risk patients showed an improved freedom from failure from 47% to 54% after a median follow up of 70 months, but showed no difference in clinical failure and overall survival [23]. Moreover, the dose constraint of 30.0 Gy to ≥ 99% of the PTV may radiobiologically have a greater impact on tumor control than the linear quadratic model suggests, especially since the validity of the linear quadratic model for extreme hypofractionation can be taken into doubt. There is substantial evidence that PCa has a low α/β of around 1.5 Gy and may therefore be more susceptible for the impact of extreme hypofractionation on tumor control [24]. Furthermore, the recent Flame trial demonstrated an advantage of an integrated focal boost of the macroscopic visible tumor in a predominantly high-risk localized PCa population in terms of biochemical recurrence free survival, without increasing toxicity [25]. It promotes the GTV as an important target for tumor control for which no concession on prescribed dose should be made, as was done in this study. Also, because of frequent tumor follow-up after treatment, recurrences will probably be diagnosed at an early stage with only localized disease. Patients then remain in the ‘window of curability’ as several salvage treatment options are available [26], [27].

To assess the effect of neurovascular-sparing treatment, we initiated a single arm phase II trial (NCT04861194) [28]. In this trial 70 men will receive neurovascular-sparing MRgRT in 5 fractions of 7.25 Gy. Because of the slight reduction in PTV dose only low- and intermediate-risk patients with a satisfactory erectile function at baseline (IIEF-5 ≥ 17) and a wish for erectile function sparing treatment are eligible. The primary endpoint is erectile function at 3 years after treatment. Secondary endpoints include biochemical recurrence free survival at 3 years after treatment and quality of life. Additionally, we will assess the dose-toxicity relationship for the individual neural and vascular structures potentially contributing to ED after radiotherapy.

The RATING guidelines for treatment planning were used for preparing the manuscript [29]. The authors concluded that the RATING score was 81%.

In conclusion, neurovascular-sparing MRgRT for localized PCa is feasible in the planning setting. Dose to the neurovascular structures can be reduced substantially. The extent of neurovascular-sparing largely depends on the patient’s GTV location.

## Role of the funding source

This research has been partly funded by ZonMW IMDI/LSH-TKI Foundation (The Hague, The Netherlands, project number 104006004), 10.13039/100011676Elekta AB (Stockholm, Sweden), and Philips Medical Systems (Best, The Netherlands). The funding sources had no involvement in the design of the study, the collection, analysis, and interpretation of the data, nor in the writing and decision to submit the article for publication.

## Declaration of Competing Interest

The authors declare the following financial interests/personal relationships which may be considered as potential competing interests: HV receives research funding from Elekta. The remaining authors declare no potential competing interests.

## Appendix A Supplementary data

Supplementary data 1

Supplementary data 2 Population-median DVH curves with 95% confidence intervals for the neurovascular-sparing 5×7.25 Gy MRgRT plans (n = 20) and the standard 5×7.25 Gy MRgRT plans (n = 20). Caption: Abbreviations: PTV = planning target volume; GTV = gross tumor volume; NVB = neurovascular bundle; NS = neurovascular-sparing; IPA = internal pudendal artery; CC = corpus cavernosum; PB = penile bulb. Femur, NVB, IPA, and CC: n = 40 (left and right side are combined); NVB constraint met in NS plan: n = 24; NVB constraint not met in NS plan: n = 16. *Standard 5×7.25 Gy MRgRT dose prescription to PTV was 34.4 Gy in ≥ 99.0% (no separate clinical target volume or GTV + 4 mm prescription). Dose constraints for bladder, rectum, sphincter, and femur were identical for the neurovascular-sparing and standard plans.
