# Supplementary material for: Adaptive magnetic resonance-guided neurovascular-sparing radiotherapy for preservation of erectile function in prostate cancer patients
Source: Phys Imaging Radiat Oncol. 2021 Sep 21;20:5–10. doi: 10.1016/j.phro.2021.09.002 (PMC8473534; doi:10.1016/j.phro.2021.09.002)
Supplement: Supplementary data 1 [file mmc1.docx]

**Supplement 1:**

Equivalent dose in 2 fractions (EQD2) and biologically effective dose (BED) of neurovascular-structure dose constraints for neurovascular-sparing radiotherapy.

| Study | Fractions | Structure | Parameter | Dose constraint (Gy) | EQD2 α/β = 2.0 Gy (Gy) | BED α/β = 2.0 Gy (Gy) | EQD2 α/β = 3.0 Gy (Gy) | BED α/β = 3.0 Gy (Gy) |
| --- | --- | --- | --- | --- | --- | --- | --- | --- |
| Spratt et al. [5] | 42 | IPA | 100% | <36.0 | 25.7 | 51.4 | 27.8* | 46.3 |
|  |  | CC | 100% | <30.0 | 20.4 | 40.7 | 22.3* | 37.2 |
| Present study | 5 | NVB | D0.1cc | ≤32.8 | 70.0 | 140.0 | 62.6 | 104.3 |
|  |  | IPA | D0.1cc | ≤20.0 | 30.0 | 60.0 | 28.0 | 46.7 |
|  |  | CC | D0.01cc | ≤17.3 | 23.5 | 47.0 | 22.3 | 37.1 |
|  |  | PB | D50% | <29.5 | 58.3 | 116.5 | 52.5 | 87.5 |

*IPA and CC 5 fraction equivalent dose constraints adapted for the present study.

Abbreviations: NVB = neurovascular bundle; IPA = internal pudendal artery; CC = corpus cavernosum; PB = penile bulb.
