# Supplementary figures and images for: Adaptive magnetic resonance-guided neurovascular-sparing radiotherapy for preservation of erectile function in prostate cancer patients
Source: Phys Imaging Radiat Oncol. 2021 Sep 21;20:5–10. doi: 10.1016/j.phro.2021.09.002 (PMC8473534; doi:10.1016/j.phro.2021.09.002)

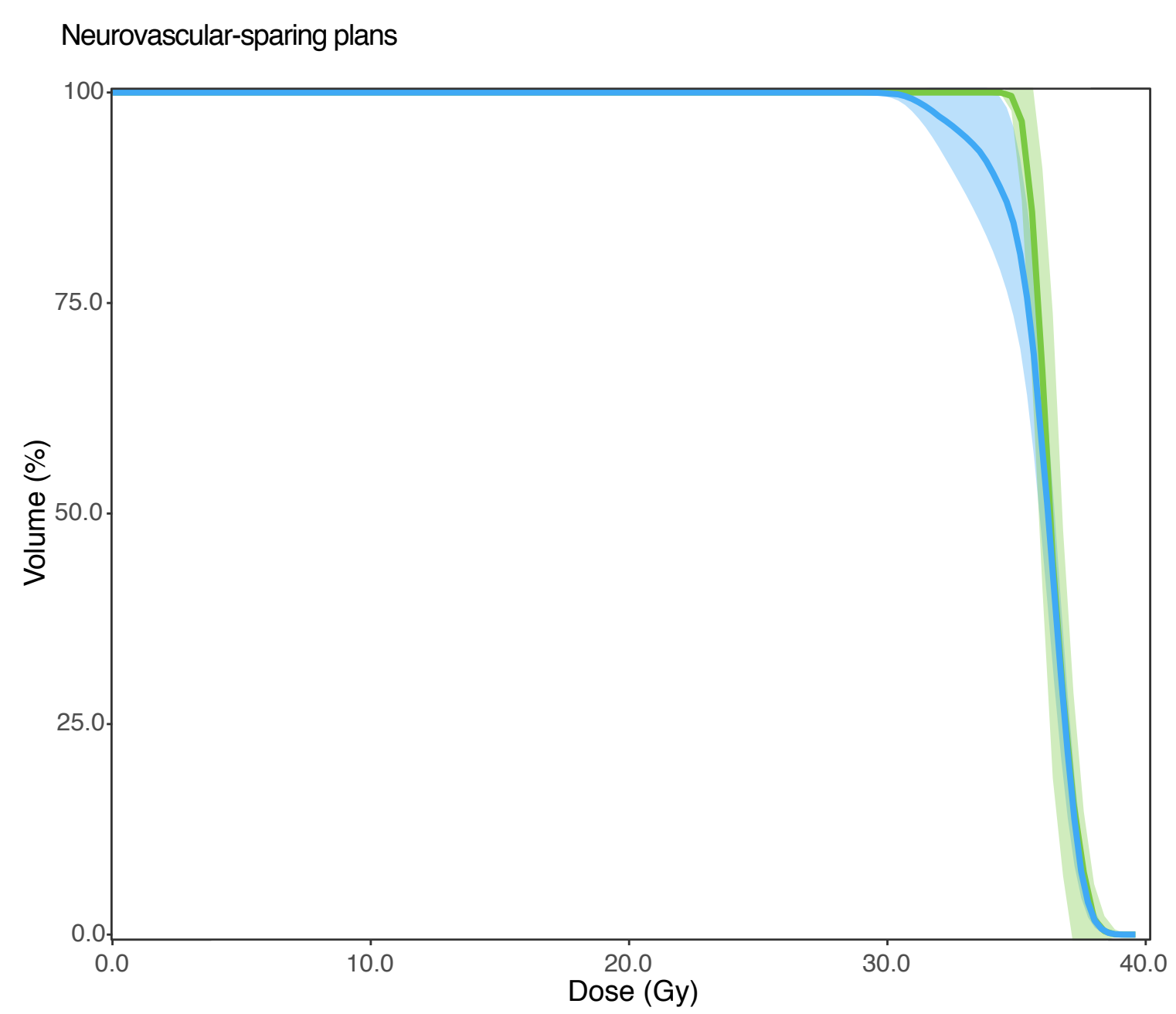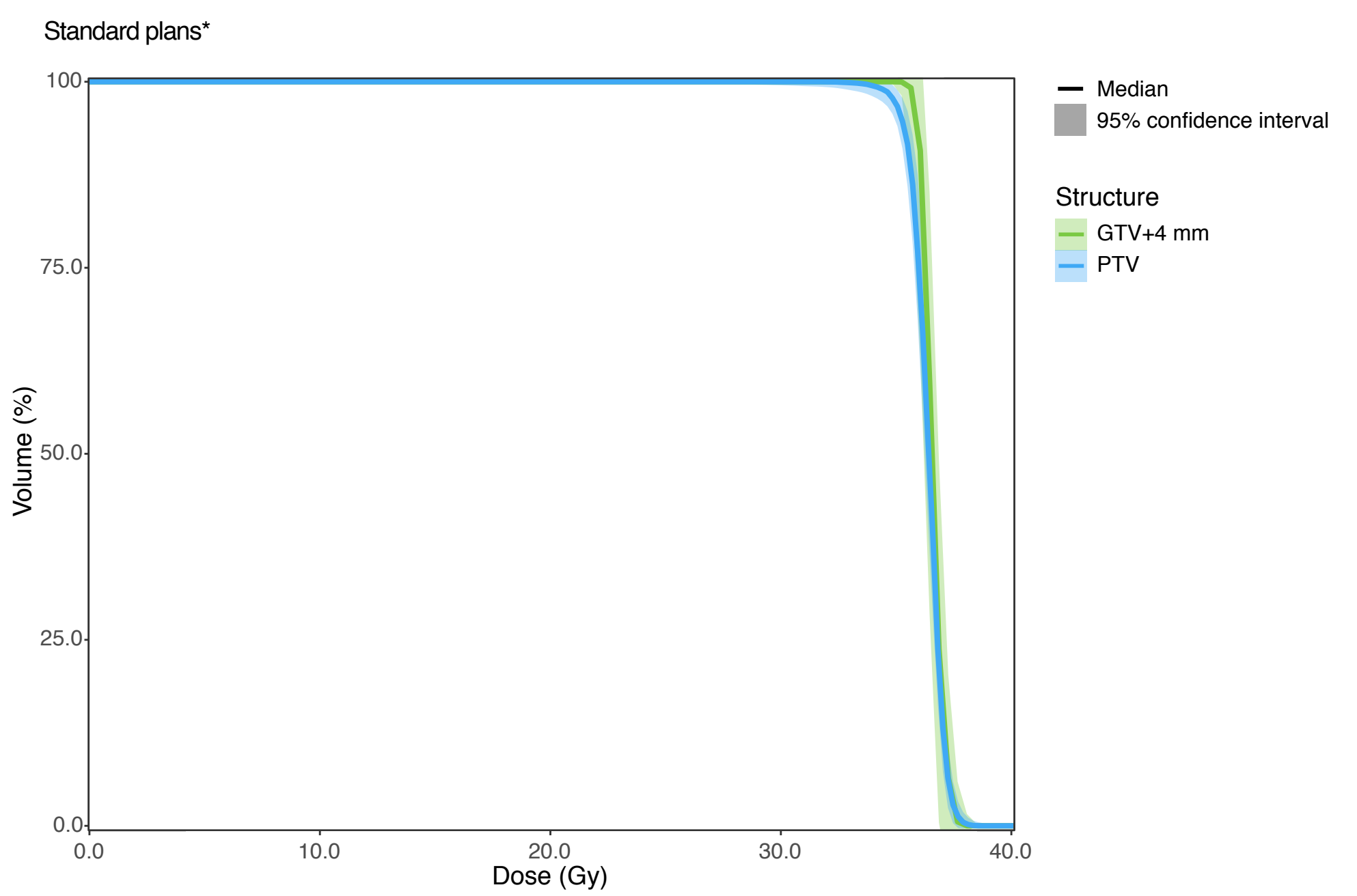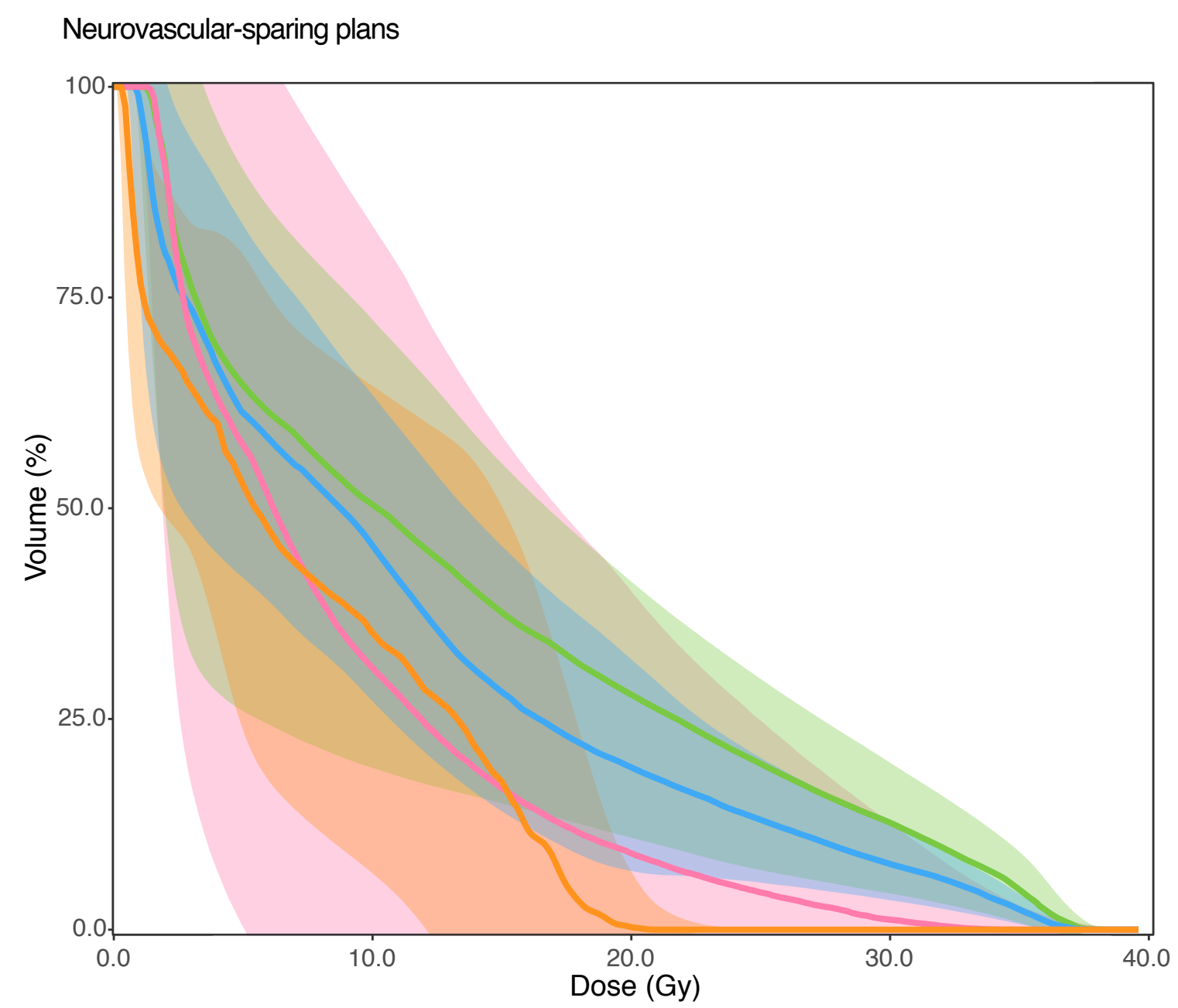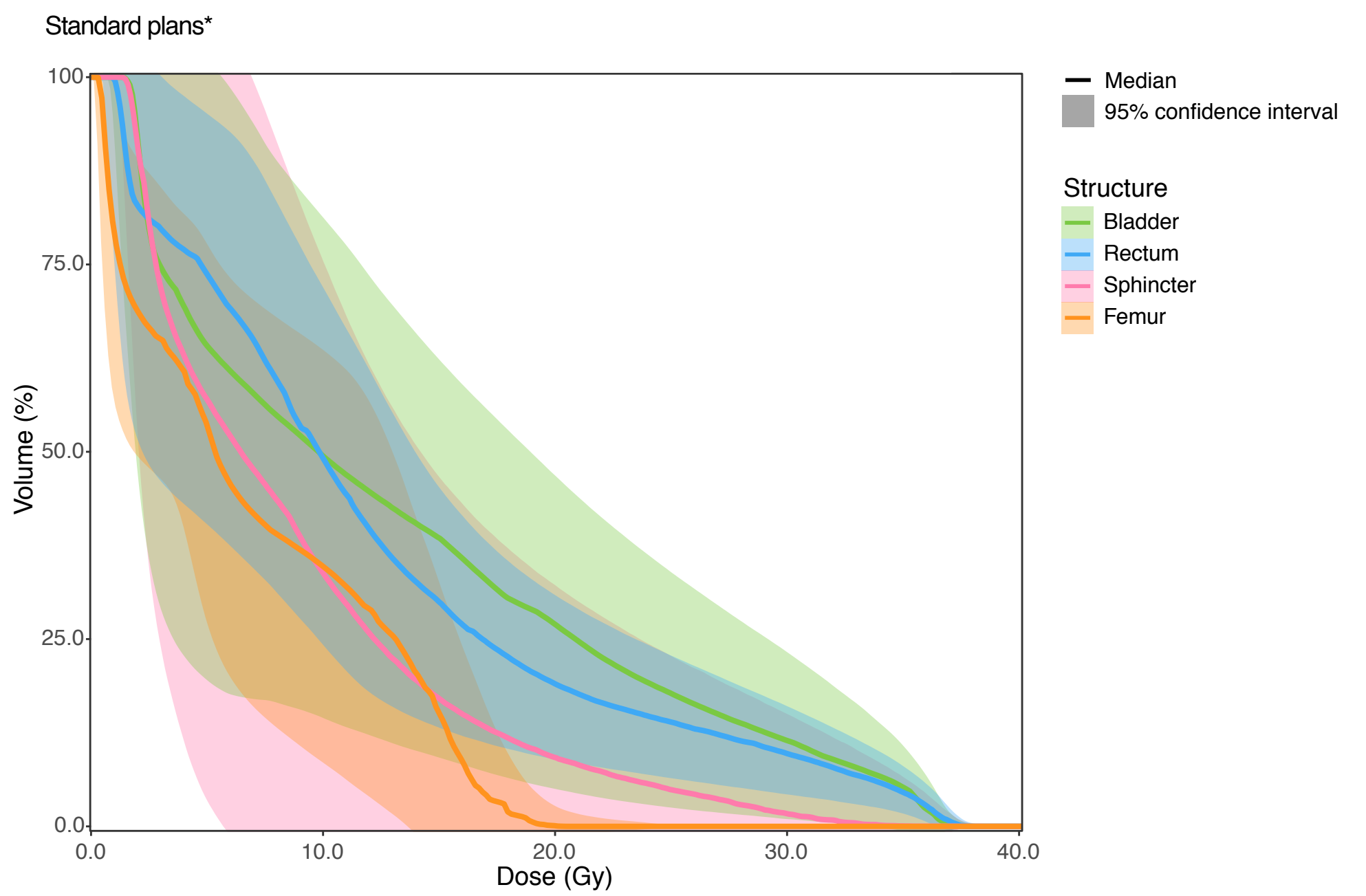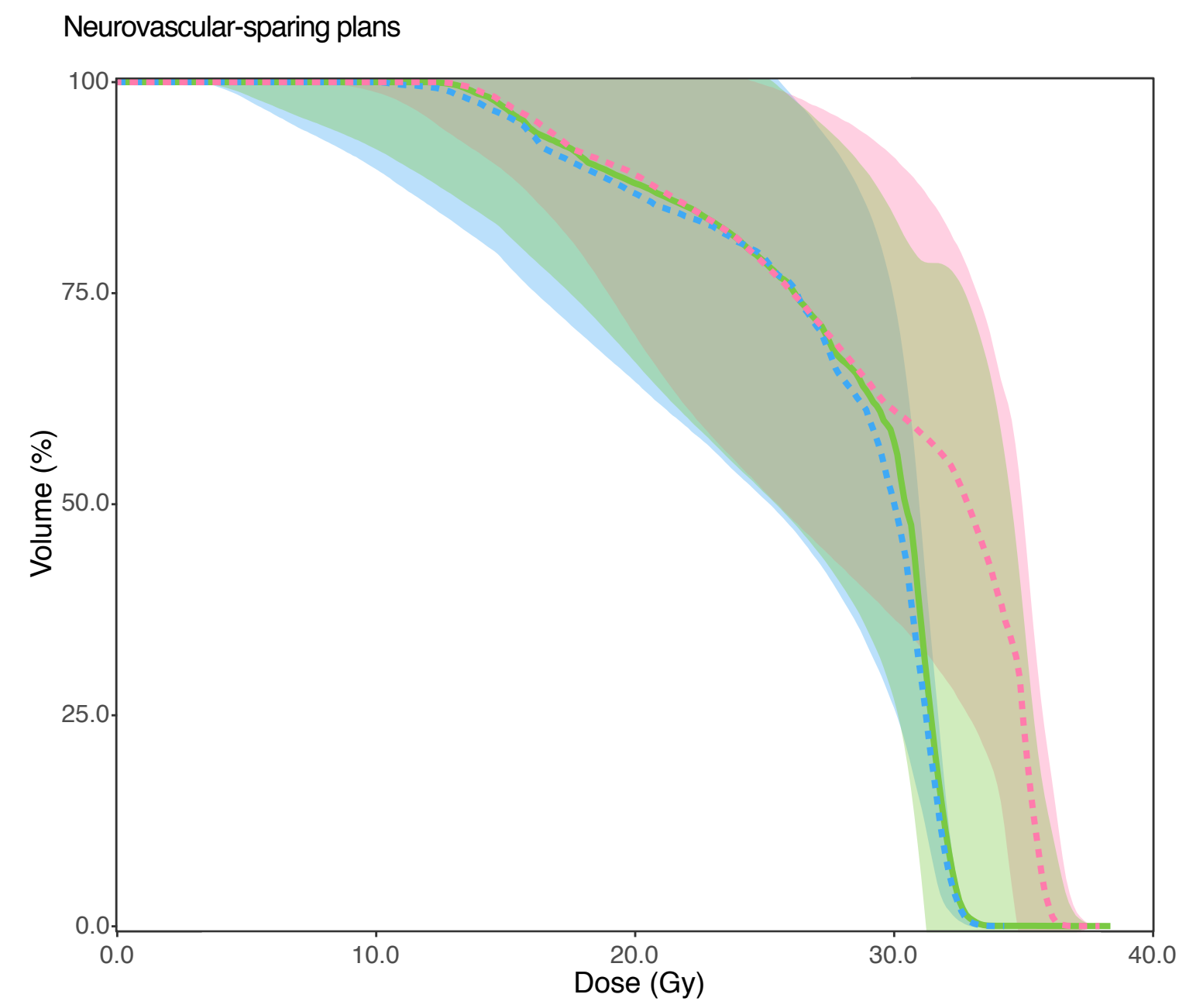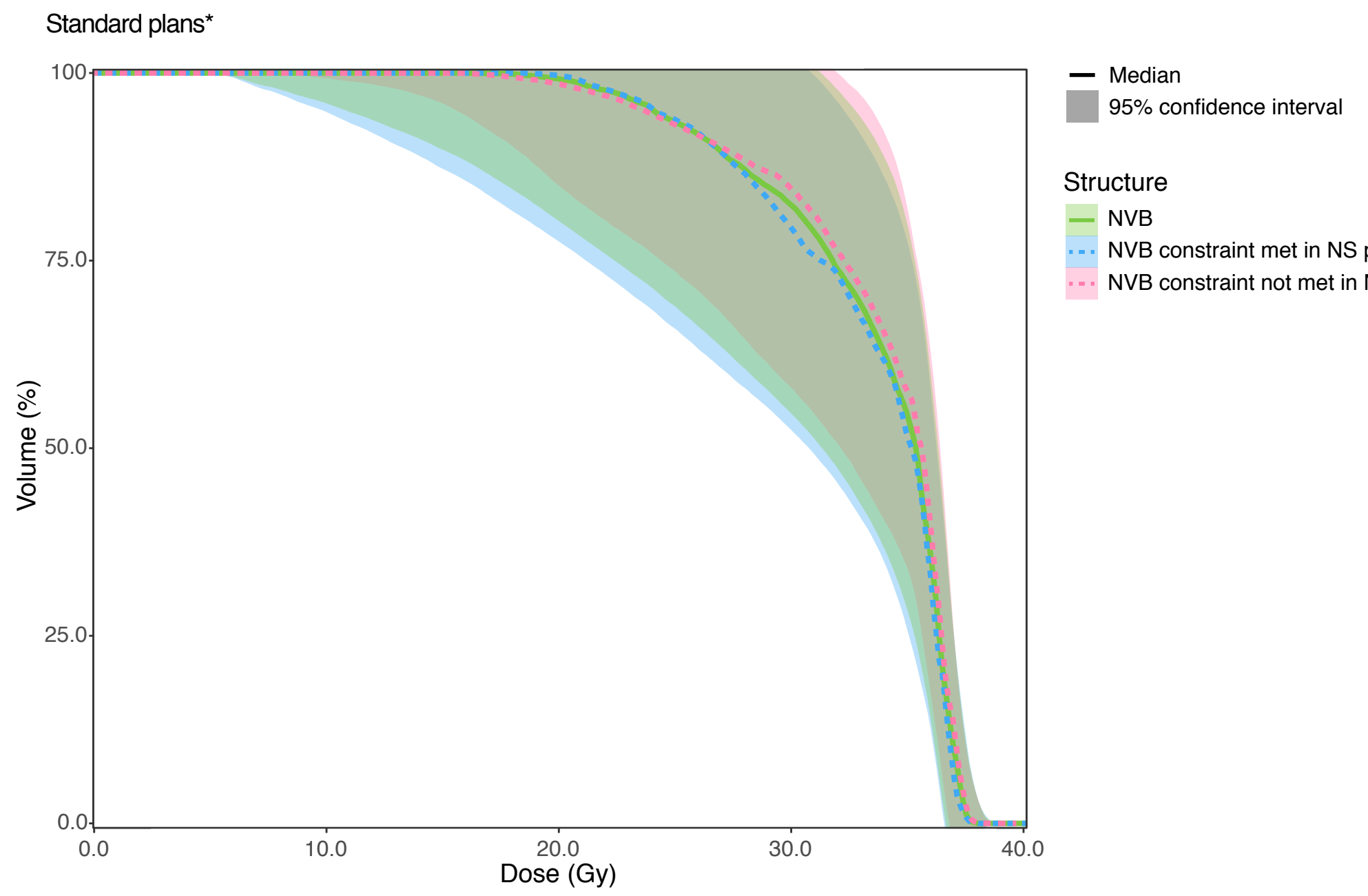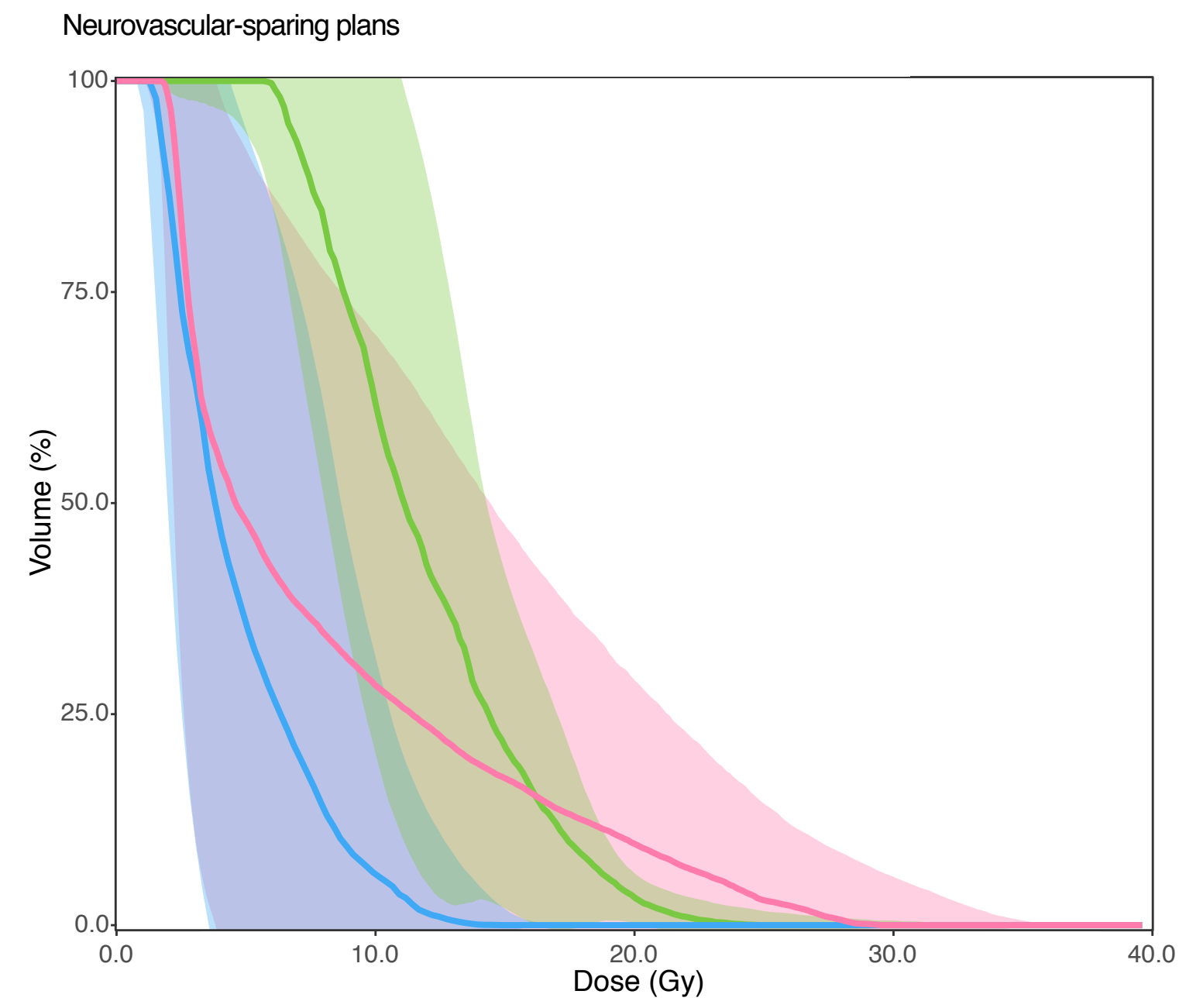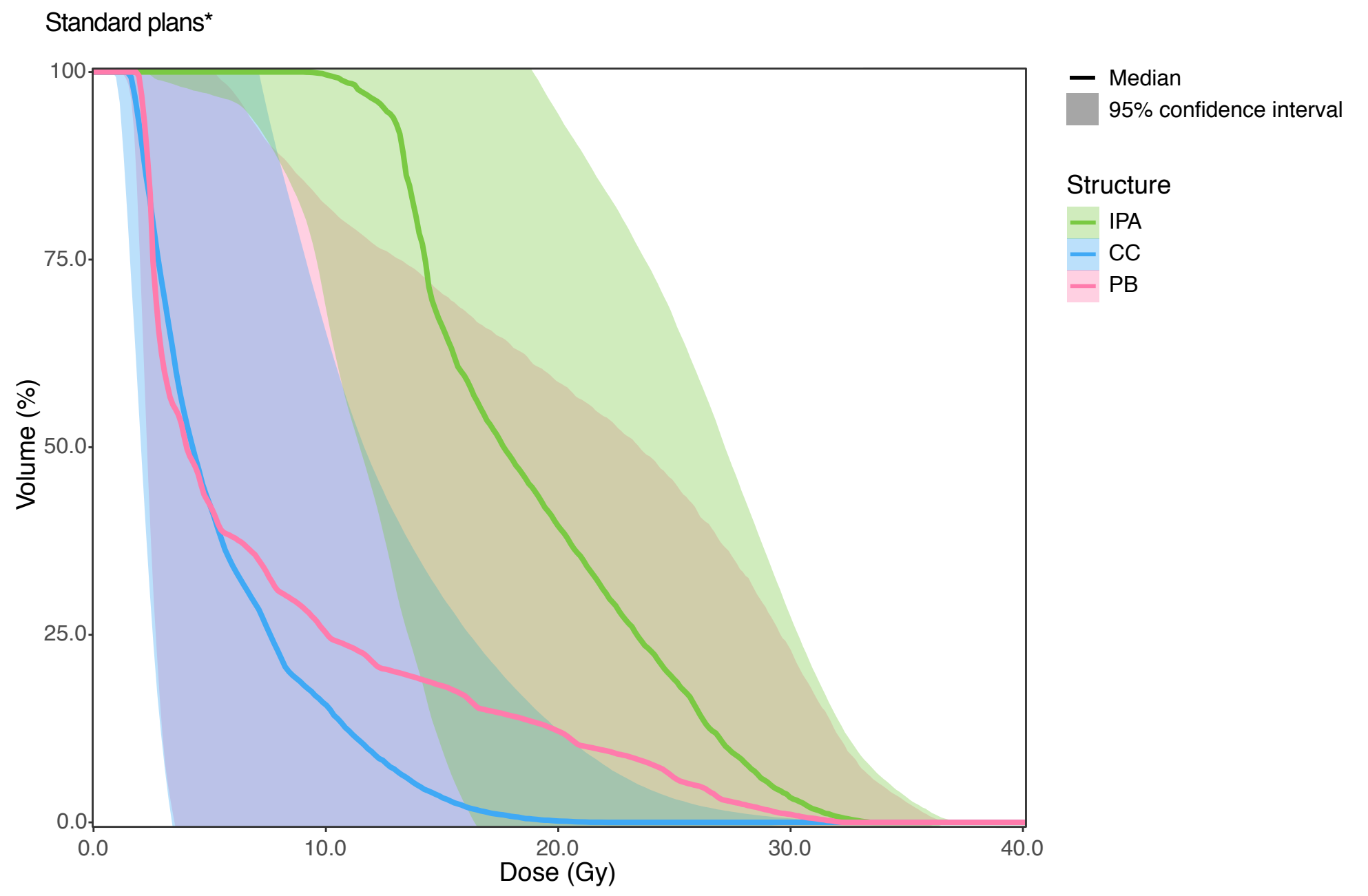

Supplement: Supplementary data 2 — Population-median DVH curves with 95% confidence intervals for the neurovascular-sparing 5×7.25 Gy MRgRT plans (n = 20) and the standard 5×7.25 Gy MRgRT plans (n = 20). Caption: Abbreviations: PTV = planning target volume; GTV = gross tumor volume; NVB = neurovascular bundle; NS = neurovascular-sparing; IPA = internal pudendal artery; CC = corpus cavernosum; PB = penile bulb. Femur, NVB, IPA, and CC: n = 40 (left and right side are combined); NVB constraint met in NS plan: n = 24; NVB constraint not met in NS plan: n = 16. *Standard 5×7.25 Gy MRgRT dose prescription to PTV was 34.4 Gy in ≥ 99.0% (no separate clinical target volume or GTV + 4 mm prescription). Dose constraints for bladder, rectum, sphincter, and femur were identical for the neurovascular-sparing and standard plans. [file mmc2.pdf]
